# Electronic properties of MoS_2_/MoO_x_ interfaces: Implications in Tunnel Field Effect Transistors and Hole Contacts

**DOI:** 10.1038/srep33562

**Published:** 2016-09-26

**Authors:** Santosh K. C., Roberto C. Longo, Rafik Addou, Robert M. Wallace, Kyeongjae Cho

**Affiliations:** 1 Department of Materials Science & Engineering, The University of Texas at Dallas, Richardson, TX 75080 USA; 2 Materials Science and Technology Division, Oak Ridge National Laboratory, Bethel Valley Road, Oak Ridge, Tennessee 37831, USA; 3 Department of Physics, The University of Texas at Dallas, Richardson, TX 75080 USA.

## Abstract

In an electronic device based on two dimensional (2D) transitional metal dichalcogenides (TMDs), finding a low resistance metal contact is critical in order to achieve the desired performance. However, due to the unusual Fermi level pinning in metal/2D TMD interface, the performance is limited. Here, we investigate the electronic properties of TMDs and transition metal oxide (TMO) interfaces (MoS_2_/MoO_3_) using density functional theory (DFT). Our results demonstrate that, due to the large work function of MoO_3_ and the relative band alignment with MoS_2_, together with small energy gap, the MoS_2_/MoO_3_ interface is a good candidate for a tunnel field effect (TFET)-type device. Moreover, if the interface is not stoichiometric because of the presence of oxygen vacancies in MoO_3_, the heterostructure is more suitable for *p*-type (hole) contacts, exhibiting an Ohmic electrical behavior as experimentally demonstrated for different TMO/TMD interfaces. Our results reveal that the defect state induced by an oxygen vacancy in the MoO_3_ aligns with the valance band of MoS_2_, showing an insignificant impact on the band gap of the TMD. This result highlights the role of oxygen vacancies in oxides on facilitating appropriate contacts at the MoS_2_ and MoO_x_ (x < 3) interface, which consistently explains the available experimental observations.

The aggressive miniaturization of silicon-based electronics, reaching a fundamental limit of scaling, has motivated the electronic device community to explore for alternative channel materials and device architectures for the future transistor technology^1^,^2^. Within this context, atomically thin two dimensional (2D) graphene and hexagonal boron nitride (h-BN) have emerged as potential candidates for device application because of the advances in exfoliation methods and synthetic techniques^3^,^4^,^5^,^6^. However, due to the lack of a sizeable band gap in graphene and the large band gap (>5 eV) of h-BN, these 2D materials have limitations in their use as channel materials in low power transistor applications. In the search for other thin 2D semiconductors with optimum electronic properties, 2D transition metal dichalcogenides (TMDs) have recently attracted a significant interest, as these materials possess sizeable band gaps (1–2 eV), ideally no dangling bonds and correspondingly low trap densities at semiconductor-dielectric interface allowing efficient electrostatics, as well as the reduction of short channel effects^7^,^8^. Moreover, TMDs provide a wide range of materials choices, and have tunable electronic and optical properties through thickness control, mixed alloys^9^,^10^, combination of TMD heterostructures, phase and strain engineering or with the application of an external electric or magnetic field^11^,^12^,^13^. Additionally, the electronic and optical properties can be modulated through the dielectric environment^14^,^15^,^16^. Single layer TMDs have a direct type band gap in the visible portion of the electromagnetic spectrum. As a result, TMDs are especially suitable for optoelectronic, digital electronics, and display devices.

However, there are still various challenges before the realization of an ideal device concept based on TMDs. Perhaps the most important is to find a suitable contact between a true metal and the TMD semiconductor with low interfacial resistance, to enable efficient charge injection and/or extraction. Thus, achieving low contact resistance for TMD-based nano-electronic devices is the critical first step in order to get a good device performance. There are reports on true metal contacts with TMDs showing several problems, including degradation of the performance of TMD transistors due to undesired interface reactions, contact resistance or posing an abnormal Fermi level pinning at the band gap of the semiconductor^17^,^18^,^19^. Moreover, recent studies have shown the importance of the contact metal deposition ambient and the resultant contact properties^20^,^21^,^22^. Therefore, alternative contact materials which can effectively facilitate the charge transfer between the source/drain and the semiconductor (channel material) need to be sought. There has also been research on stable doping strategies to lower the contact resistance^23^,^24^, and different options, like transition metal oxides, have been used as a barrier layer between metals and organic semiconductors in organic photovoltaics (OPVs) devices for selective charge transfer^25^. However, utilizing a metal oxide contact layer with transition metal dichalcogenides has not been realized until recently^26^,^27^,^28^. Hence, the specific nature of their interfacial electronic properties must be accurately determined before pursuing further extensive research on TMDs-metal oxide contacts.

Another interesting possibility is given by the corresponding TMD-TMO interfaces. From this point of view, Molybdenum trioxide (MoO_3_) could be considered as a promising hole contact on MoS_2_-based Field Effect Transistors (FET). MoO_3_ is stable in ambient conditions, and it provides an efficient hole extraction. Unlike true metal contacts, it does not induce Fermi level pinning at the interface. Our own work has previously shown that the interaction with metals at metal-TMD interface modifies the transition metal-chalcogen hybridization of the TMD, inducing states in the band gap, which ultimately results in unusual Fermi level pinning^17^. Accordingly, there is an urgent need to find a suitable contact material or barrier layer that could also avoid Fermi level pinning, resulting in a nearly Ohmic contact. Currently, TMD-based devices are measured with Schottky limited electrical characteristics^23^. A good contact would facilitate the electron injection and extraction during the device operation.

In this paper, we present an investigation on the atomic structures and electronic properties of MoS_2_/MoO_x_ (x ≤ 3) interfaces for future FET-based device applications^26^,^27^,^28^. Our results show that TMD and MoO_x_ interfaces enable ideal *p-*type contact characteristics for future transistor technology. Moreover, due to defect-induced gap states (i.e., defect bands) in sub-stoichiometric MoO_x_, the interface presents an Ohmic character, which may result in a promising Ohmic contact for TMD-based electronic devices. Therefore, this work provides a fundamental understanding of the interfacial electronic properties of MoS_2_ and both pristine and oxygen deficient MoO_x_ (x ≤ 3).

## Methodology

First-principles calculations based on Density Functional Theory (DFT)^28^,^29^,^30^,^31^ with plane wave basis set and Projector Augmented Wave (PAW)^32^,^33^ pseudopotentials have been performed using the Vienna *Ab-initio* Simulation Package (VASP)^33^,^34^,^35^. The electronic wave functions were represented by plane wave basis with a cutoff energy of 500 eV and the exchange correlation interactions were incorporated as a functional of the Generalized Gradient Approximation (GGA)^34^. Additional calculations included the hybrid Heydt-Scuseria-Ernzerhof (HSE) exchange- correlation functional^36^ and many body methods (GW0)^37^. In order to investigate the MoS_2_/MoO_3_ contact interface, a supercell structure with a S-terminated MoS_2_ (001) surface and a O-terminated MoO_3_ (010) surface was constructed. The lattice mismatch for such superstructure is less than 1% (the strain is on the MoO_3_ surface). Periodically repeated slabs separated by a 16 Å thick vacuum region were used, in order to avoid the interaction between the two surfaces of the slab as a result of the periodic boundary conditions (PBC). During the calculations, the atoms were allowed to relax while the cell size was kept fixed. A Г-centered 6 × 6 × 1 and a 12 × 12 × 1 *k*-point meshes were then used for the self-consistent field (SCF) and density of states (DOS) calculations, respectively. The energy and forces were converged until a tolerance value of 10^−5^ eV and 0.01 eV/Å, respectively. Because standard DFT is unable to grasp the physics of van der Waals (vdW) interactions leading to overestimating the interlayer separation in layered materials, the Grimme vdW-D2 approach^38^,^39^ was adopted in order to optimize the MoS_2_ and MoO_3_ interlayer distance accurately.

## Results and Discussion

**Figure 1:**
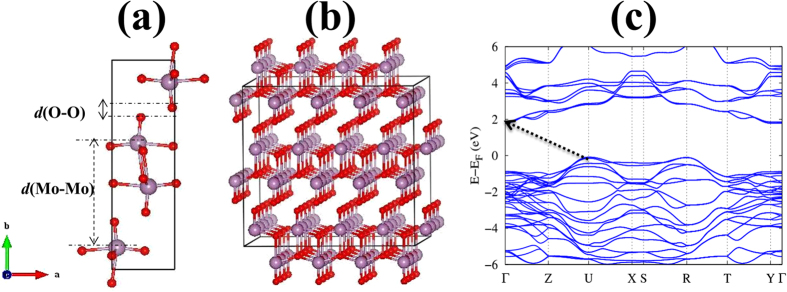
(**a**) Side view of the atomic structure of the MoO_3_ unit cell. The interlayer metal to metal distance and the vdW gap are indicated by *d*(Mo-Mo) and *d*(O-O), respectively. (**b**) The MoO_3_ 4 × 1 × 4 supercell used for the defects study, showing the layered structure along the [010] direction. Red and purple spheres represent O and Mo atoms, respectively. (**c**) Electronic band structure of bulk MoO_3_, showing the indirect band gap (indicated by an arrow) with the CBM at Г (0.0 0.0 0.0) and the VBM at U (0.5 0.0 0.5) high-symmetry k-points.

**Figure 2:**
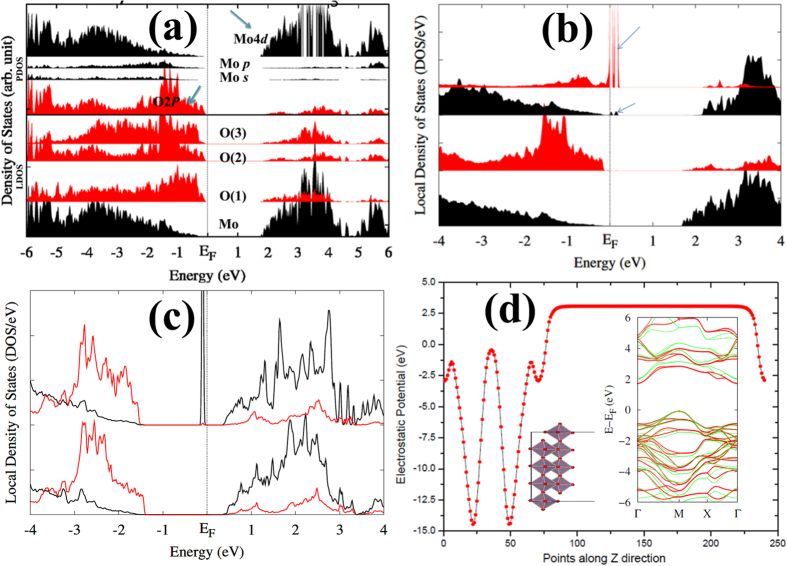
DOS for bulk MoO_3_ in (**a**) pristine form, (**b**) with Mo vacancy (V_Mo_), and (**c**) O vacancy (V_O_). Red, and black lines represent O and Mo DOS, respectively. Gap states caused by defects are indicated by arrows. (**d**) The electrostatic potential variation along the [010] direction is shown for a single MoO_3_ layer. The inset shows the atomic and electronic band structures for monolayer.

We first investigate the electronic properties of bulk and single layer Molybdenum-trioxide (MoO_3_) in detail. MoO_3_ shows two phases (α and β): α-MoO_3_ is stable in an orthorhombic crystal structure with space group Q_h_^26^ (*Pbnm*) (the unit cell lattice parameters are *a* = 3.962 Å, *b* = 13.855 Å, and *c* = 3.699 Å), and the β phase is observed only at high pressure and is metastable at ambient conditions^40^,^41^. In the α-MoO_3_ phase (the one considered in this work), each unit cell contains four MoO_3_ formula units and has an easy cleavage (010) plane, as shown in Fig. 1(a,b). Each monolayer consists of two sublayers, with periodically arranged MoO_6_ octahedra. Thus, the crystal structure contains three distinct oxygen atoms due to their different coordination: asymmetrical bridging oxygen (unequal bond length with Mo), symmetric bridging oxygen (two Mo bonds with the same bond length and an elongated bond to the next sublayer), and terminal oxygen (single bond Mo-O). The terminal oxygen atom is preferentially deficient during an exfoliation process. The interlayer metal to metal distance *d* (Mo-Mo) is ~7.00 Å (the Mo-Mo distance within the same layer is 4.03 Å), and the effective vdW gap, *d* (O-O), is ~0.799 Å (See Fig. 1(a)). The electronic band structure for bulk MoO_3_ is shown in Fig. 1(c), which indicates that the band gap is of indirect type with the valance band maximum (VBM) at U (0.5 0.0 0.5) and the conduction band minimum (CBM) at Г (0.0 0.0 0.0) point. Our obtained band gaps (E_g_) are 1.9 and 2.7 eV at the GGA and HSE levels of calculation, respectively. The HSE result is closer to the experimental values of 3.2 eV for bulk and 2.8 eV for polycrystalline MoO_3_, as obtained from absorption spectra measurements^40^. As can be seen in the DOS shown in Fig. 2(a), the MoO_3_ CBM is mainly contributed by Mo *d* states and the VBM by O *p* states.

Oxygen vacancies can be easily created by ion bombardment or loss of preferential bridging oxygens from reactions and/or annealing. Such oxygen vacancies may cause surface reconstruction depending on their concentration, by turning exposed Mo atoms into chemically active sites and participating in the adsorption of new species or MoO_x_ reconstruction. MoO_x_ has a very large work function (~6.67 eV from our DFT calculations, close to available experimental values^26^,^42^), compared to the work function of metals^43^ For defective, MoO_x_, we have computed the formation energy (*E*^*form*^ = *E*^*defect*^ − *E*^*perfect*^ + *μ*_*x*_, where, μ: chemical potential of *x* = Mo and O) of Mo and O vacancies. Our results show that the O vacancy (V_O_) is energetically more favorable (by ~2 eV) than the Mo vacancy (V_Mo_). Figure 2(b,c) show the corresponding DOS. Both defects induce gap states in the MoO_3-x_ band gap. However, the Mo vacancy induces multiple gap states (of O *p* nature) close to the VBM (see Fig. 2(b)) due to unsaturated oxygen atoms, whereas an O vacancy leaves a Mo dangling bond, causing defect states of Mo *d* nature close (0.56 eV) to the CBM, as shown in Fig. 2(c). The defect level shifts downwards by 0.29 eV when using HSE. However, its electronic nature is not modified (*i.e*., its relative position in the band gap is not changed).

Similar to MoS_2_, MoO_3_ shows strong intralayer chemical bonds (Mo-O) and a relatively weak van der Waals interlayer interaction, which facilitates the exfoliation. Since MoO_3_ layers are stacked along the [010] direction, they can be easily cleaved and exfoliated to produce a thin quasi-2D crystal. The exfoliated monolayer of MoO_3_ contains two layers of Mo atoms with a thickness of ~1.38 nm (the lattice parameters of the single layer unit cell are *a* = 0.39 nm, *b* = 0.613 nm, *c* = 0.36 nm). From bulk to monolayer MoO_3_, the change in the electronic properties is not as significant as in the case of MoS_2_. Figure 2(d: inset) shows the electronic band structure of monolayer MoO_3_ (010). The obtained band gaps are E_g_ = 1.804 eV (GGA), E_g_ = 2.097 eV (GGA + U), E_g_ = 2.86 eV (HSE) and E_g_ = 3.66 eV (GW0). The gap is always indirect irrespective of the thickness, unlike MoS_2_ which shows a direct-indirect transition with decreasing thickness. The conduction band edge is dominated by Mo *d* orbitals and the valance band edge is mainly contributed from O 2*p* states, as in the bulk case. The work function (ɸ = E_vac_ − E_F_; where E_vac_ is the vacuum level and E_F_ the Fermi level) is also estimated to be ~6.6 eV from our DFT calculations, close to the experimental value^26^,^42^.

**Figure 3:**
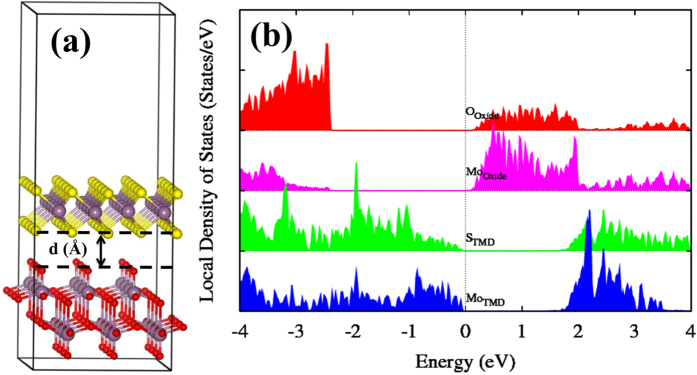
(**a**) Atomic structure of the stoichiometric MoS_2_/MoO_3_ interface. Red, purple and yellow spheres represent O, Mo and S atoms, respectively. The interlayer distance was optimized using the DFT + vdW approach. (**b**) The corresponding DOS of the interface. Green and blue lines represent the DOS of S and Mo atoms from the MoS_2_ layer, whereas red and pink lines correspond to the O and Mo atoms from the MoO_3_ layer.

In order to investigate the electronic properties of the MoS_2_/MoO_3_ interface, a heterostructure using the MoO_3_ (010) and MoS_2_ (001) monolayer surfaces was constructed and subsequently optimized. If any defects are present, the electronic properties of the pristine MoS_2_ (001) monolayer will be altered significantly and a surface passivation treatment will be crucial before constructing the interface^44^,^45^,^46^. Figure 3(a) shows the atomic configuration of the MoS_2_/MoO_3_ interface model. As stated previously, the corresponding interlayer distance optimization was done using the DFT + vdW approach, to account for the weak MoS_2_-MoO_3_ interaction. Our calculations show that standard DFT overestimates the interlayer distance by ∆*d* ~1.7 Å, with the obtained DFT + vdW result being *d*(S-O) ~2.8 Å. The DOS of the optimized interface model reveals the relative band alignments between both monolayers. The overall band gap of the pristine MoS_2_/MoO_3_ stack is substantially reduced (~0.22 eV) with respect to the respective separate counterparts, due to the metal oxide empty states appearing in the band gap energy range of MoS_2_. Including the HSE correction, the interface band gap only increases to 0.51 eV, even though the gaps of the individual monolayers widen significantly. As shown in Fig. 3(b), the VBM of MoS_2_ is located close to the CBM of MoO_3_.

Then, the conduction bands of MoO_3_ are lowered relative to those of MoS_2_, thus making a negative conduction band offset (CBO); this will result in the population of the upper MoO_3_ energy level from the MoS_2_ Fermi level. Mo *d* orbitals hybridized with O *p* orbitals from the MoO_3_ layer dominate the CBM, with MoS_2_ Mo *d* and S *p* hybridized orbitals dominating the VBM. The relatively small band gap makes this junction a promising contact heterostructure for tunnel field effect devices, where the gap, the band alignment and the interband charge carrier tunneling can be further controlled by an external applied field and the gate bias.

**Figure 4:**
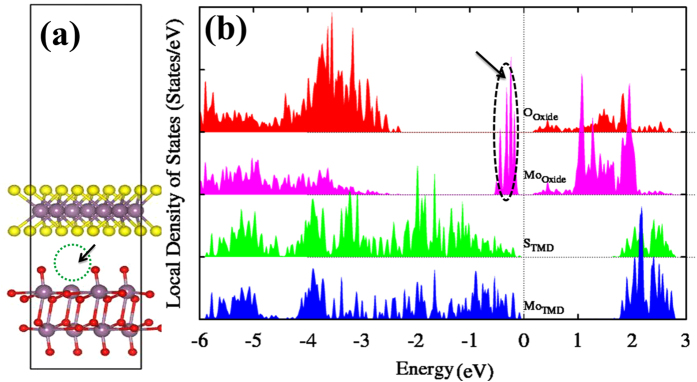
(**a**) Side view of the atomic structure of a non-stoichiometric MoS_2_/MoO_x_ interface model. The oxygen vacancy site is indicated by an arrow pointing to a circle. Red, purple and yellow spheres represent O, Mo and S atoms, respectively. (**b**) The corresponding DOS of the defective interface. The green and blue filled lines represent the DOS of S and Mo atoms from the MoS_2_ layer, whereas red and pink filled lines corresponds to the O and Mo atoms from the MoO_x_ layer. The arrow indicates the gap states caused by an oxygen vacancy in the MoO_3_ layer.

MoO_3_ is thought to contain a certain amount of oxygen vacancies (up to 3%), and is known to behave as a *p*-type contact with TMDs or other semiconductors^26^. Therefore, a model interface structure with oxygen vacancies was also investigated, in order to examine the effect of possible O vacancies on the electronic properties. Indeed, our calculations show that the MoO_x_ interfacial layer can behave as a *p-*type contact. Moreover, a unique band alignment between (MoS_2_/MoO_x_) with an almost zero charge injection barrier is formed, as demonstrated by the DOS shown in Fig. 4(b). The MoS_2_/MoO_x_ contact does not cause Fermi level pinning, showing a superior contact performance over other true metals^17^,^18^,^19^. Therefore, our results clarify why the defective MoS_2_/MoO_x_ interface can also be used as an ideal hole contact for TMD-based devices, besides the well-studied organic semiconductors. The presence of O vacancies produce Mo dangling bonds, which induces defect gap states in the upper region of the MoO_x_ band gap (close to the CBM). Moreover, this is an extraordinarily localized effect with only slight changes showed in the electronic structure of the neighboring atoms. These defect states of Mo 4*d* nature are just empty states that can be easily filled by electron transfer from the valance band of the MoS_2_ layer, creating the *p-*type MoS_2_. In other words, it behaves as a MoO_x_ hole contact layer, injecting holes into the MoS_2_.

Therefore, MoO_x_ has a great potential to be used as an efficient hole injection layer by charge transport through the valence band, thus making a TMD-based *p-*FET. The overall performance of a hypothetical FET electronic device depends on the metal/MoO_x_ and MoO_x_/TMD contacts. However, the metal/MoO_x_ contact resistance can be properly tuned with a suitable choice of metal with a specific work function^43^. On the contrary, MoO_x_/MoS_2_ contacts have been regarded as a major hurdle for many years. Recent experimental reports have shown a low hole Schottky barrier in a device study utilizing MoO_x_ contacts with MoS_2_ and WSe_2_ TMDs^26^.

Due to the relatively large difference between the VBM and the CBM of the MoO_x_, the band alignment with MoS_2_ should also be very suitable for tunneling device applications. MoO_x_ films were also found to be oxygen-deficient in a nitrogen environment, enhancing the device performance by means of a gap state mechanism^47^ which is consistent with the results of this work. Furthermore, by changing the thickness of MoO_x_ and MoS_2_ layers, additional broad gap states can be introduced, which would substantially increase the hole transport^48^.

The interfacial electronic transport can also be modulated with an applied external electric field. However, synthesizing ideal or completely stoichiometric MoO_3_, or having controlled oxygen vacancy formation is actually a challenge. Thus, the contact is better realized due to the presence of oxygen vacancies in the MoO_3_. Interestingly, the formation of self-limiting WO_x_ (x < 3) on atomically thin WSe_2_ was used as charge transfer dopants and low hole Schottky barrier contacts for *p-*WSe_2_ transistors^49^. Hence, given the numerous possible transition metal oxides with a wide range of work functions and electronic structures, and the additional available options to tailor their properties, such as modifying the defect concentration or the cation oxidation states, this research focused on hole contacts based on metal oxides-TMD heterostructures, and opens a new avenue to explore 2D TMDs and TMO interfaces for optimization of the device functionality.

### Summary

The electronic properties of the sub-stoichiometric MoO_x_/MoS_2_ interface have been investigated using DFT calculations. Our results reveal that, due to the large work function of MoO_3_, a unique and useful band alignment between MoS_2_ and MoO_3_ is observed, which can open a potential application in FET. Moreover, our findings are in excellent agreement with experimentally-observed sub-stoichiometric MoO_x_ that shows a defect level alignment with the valance band of the TMD, creating an Ohmic-type contact perfectly suitable for metal-semiconductor device contact purposes. This result also indicates that the MoO_x_/MoS_2_ interface facilitates spontaneous charge transfer from the TMD to MoO_x_ and *vice versa*. In other words, the defect state in the band gap of MoO_x_ assists the hole injection at the interface (through the formation of a low hole injection barrier, thus facilitating an Ohmic injection). This theoretical work sheds light on the atomic level mechanisms of TMD/TMO interfaces, showing their promising characteristics for semiconductor device applications.

## Additional Information

**How to cite this article**: KC, Santosh *et al*. Electronic properties of MoS_2_/MoO_x_ interfaces: Implications in Tunnel Field Effect Transistors and Hole Contacts. *Sci. Rep.* **6**, 33562; doi: 10.1038/srep33562 (2016).
